# Suitability of Blending Rice Husk Ash and Calcined Clay for the Production of Self-Compacting Concrete: A Review

**DOI:** 10.3390/ma14216252

**Published:** 2021-10-20

**Authors:** Abubakar Muhammad, Karl-Christian Thienel, Ricarda Sposito

**Affiliations:** Institute for Construction Materials, University of the Bundeswehr Munich, 85577 Neubiberg, Germany; christian.thienel@unibw.de (K.-C.T.); ricarda.sposito@unibw.de (R.S.)

**Keywords:** self-compacting concrete, metakaolin, calcined common clays, rice husk ash, rheology, compressive strength, durability, creep, shrinkage

## Abstract

One principal approach to achieve self-compacting properties is the increased amount of finer constituents of the mixture. This, in turn, increases cement consumption leading to higher greenhouse gas emissions. Pozzolanic materials, like rice husk ash or calcined highly kaolinitic clays, have gained increased attention as supplementary cementitious materials in self-compacting concrete production. These materials could be viable alternative supplementary cementitious materials for sub-Saharan Africa which already lacks fly ash, slag and silica fume. This current effort reviews the impact of rice husk ash and calcined clays for the production of self-compacting concrete. Special focus is on their impact on rheological, mechanical and durability properties of self-compacting concrete. Rice husk ash and, in particular, calcined highly kaolinitic clays are introduced as technical and cost-effective supplementary materials for use in self-compacting. The review disclosed a lack of knowledge when it comes to the use of low-kaolinitic calcined clays as sole SCM or together with rice husk ash, which could be a very promising combination for e.g., several countries in Africa. Further studies are needed on the rheological properties, shrinkage, creep, and durability of self-compacting concrete produced with other calcined common clays and their blend with rice husk ash.

## 1. Introduction

The potential of various pozzolanic materials as partial replacements for cement in self-compacting concrete (SCC) production was established in previous studies. In order to continue with the trend of research on the use of rice husk ash (RHA) and metakaolin—one of the calcined clays—as a supplementary cementitious material (SCM) for SCC production, and to point out the missing gaps for further studies, the following review starts with introducing the concept of SCC and the SCM for its production, then a brief overview is given of the characteristics of rice husk ash and calcined clays as pozzolanic SCM. It is followed by a detailed literature study focusing on the fresh, mechanical, and durability properties of SCC with RHA, calcined clays, or a blend of both materials. 

In reinforced concrete, a sufficient concrete cover usually serves to protect the reinforcement from adverse weather effects, harmful substances, and to improve durability. A dense microstructure can only be achieved if the concrete flows properly and embeds the reinforcement closely, fills all gaps and corners of formwork without any kind of obstruction. To achieve these attributes, concrete that requires very little compaction only was first produced and used in Europe in the early 1970s [1]. At that time, the concept of self-compacting concrete (SCC)—without the application of external vibration nor compaction—was still an imaginary vision. It was first proposed in 1986 and produced at the University of Tokyo, Japan, in 1988 [2,3,4].

The flow of SCC, measured as the total spread of the mixture particles purely under the influence of gravity, is normally achieved by controlling the water–powder ratio and applying high range water-reducing admixtures (HRWR) [5]. Besides the water–powder ratio, the proportion of finer and coarser particles and the application of viscosity modifying agents (VMAs) in the mix design are decisive for the segregation resistance of SCC [3,6]. 

SCC can be classified into three types. The powder type: here, the fluidity of SCC is achieved by a reduction in coarser aggregate content and the addition of high-range water reducers. While the segregation resistance is achieved by increasing the percentage of fines [2,3,7]. This type is usually adopted for higher grades of SCC with a water-powder ratio of as low as 0.35. The VMA type: in this method, viscosity modifying agents are applied to provide the required segregation resistance of the SCC. Also, a small amount of HRWR is added to provide fluidity for the mixture [2,8,9]. This method is suitable for low grades of SCC with a water–powder ratio of 0.45. The combined type: this combines the use of VMA and increasing the percentage of powder, in controlling the segregation and fluidity of SCC. The combined type is usually employed for medium grades of SCC with the water–binder ratio of 0.4 [8,10].

The optimization of mix design towards self-compacting properties leads to superior qualities over conventional, vibrated concrete [4]. The modified flowing properties and segregation resistance both yields high homogeneity [10], a reduction of voids and higher early strength values [10,11,12], an improved interfacial transition zone, and hence to higher durability [13,14]. Also, the use of SCC improves the construction environment as the absence of concrete vibrators reduces noise pollution [1]. 

Despite its technological advantages, the market share of SCC is small due to some obstacles especially in developing countries [15,16]. First, SCC mixes are more sensitive even to a minor variation in constituent’s proportions [16,17], change in materials properties [18,19], and the production method adopted [16,20]. Secondly, and eventually the most important concern of SCC, is the high cost of its production due to the use of high dosages of chemical admixtures and the higher binder content [21]. The cost of materials in SCC is exceeding approximately 20–50% those for conventional vibrated concrete (CVC) [15]. Although, this can be partially compensated by rapid and easy placing [22]. 

Mix design of SCC entails either a large reduction of coarse aggregate and an increased powder content [3,23] or a small reduction in the coarse aggregate content and the addition of VMAs [24]. From a technological point of view, a lower content of coarse aggregates in SCC might result in a lower modulus of elasticity, which may affect negatively time-dependent deformation (creep and shrinkage) of SCC [25]. Raising the binder content, furthermore, increases the environmental impact. The method to achieve SCC with small changes in the coarse aggregate and the use of VMA, instead, leads to altered mechanical properties of the concrete [25]. To provide solutions to these problems, researchers have focused on the potentials of supplementary cementitious materials (SCM) in SCC production.

The most commonly used SCM in SCC production include fly ash [24,26,27], silica fume [27,28,29], rice husk ash [10,30,31] and metakaolin [32,33,34]. These materials yielded positive results at optimal dosage levels [10,14,24,27,33]. Out of these four materials two will not be considered in this review. First, fly ash production decline with the reduction of coal-combustion which is enforced in order to limit the global CO_2_ release. Right now, it is already hardly available in Africa or South America [35]. Second, the replacement-level of silica fume is limited due to its high water demand and portlandite consumption as well as for economic reasons.

Calcined clays and agro-waste materials could be an alternative source of SCM to sub-Saharan Africa [36]. This includes among others limestone powder [37,38], which is present in abundance across the region, clay minerals containing kaolinite and other mineral assemblage which can be calcined to a SCM [39,40,41]. The most widely used SCM from calcined clay in the region is metakaolin. However, the availability and potentialities of other clay minerals not rich in kaolinite have also been established [40,41]. Additionally, the fact that Agriculture is one of the leading economic sector of the sub-Saharan Africa, agricultural waste such as RHA, palm oil fuel ash, cassava ash, bagasse ash, bamboo leaf ash, corn cob ash, are another viable source of SCM present in a significant amount to be used in concrete [21,36,42,43].

Rice husk ash contains about 90% reactive amorphous silica making it suitable to be used as a pozzolanic material. At cement replacement level of 15%, RHA was found to improve the microstructure of the interfacial transition zone between the cement and the coarse aggregate and, as a consequence, the strength and durability of SCC [31]. Further studies revealed improved mechanical properties of SCC with RHA as a substitute to VMA [10]. The use of RHA as both substitutes to VMA and partially to cement could cut the cost of SCC production by approximately 40% [10]. This may probably be true but it cannot be generalized due to variation in market forces, quality of the RHA itself, and the type of other binders used. However, the use of RHA in SCC requires an increased dosage of superplasticizers and causes a reduction in early strength [44]. 

Pozzolanic materials for SCC can also be obtained from common clays containing different phyllosilicates (kaolin, montmorillonite, illite, etc.) when thermally activated [30,45,46,47,48,49]. Similarly, it can also be obtained from clay wastes mostly obtain from dumps sludge, and water treatment plants [50], or further origin of burned clays such as clay bricks [51], ceramics, and tiles [52]. 

The use of metakaolin as cement replacement enhanced the precipitation of C-S-H leading to the refinement of specific pore size distribution of concrete, and densification of its microstructure [45,46,53,54]. These attributes make it the most commonly used calcined clay in SCC. On the other hand, the use of metakaolin increases the water demand of cement paste and the heat of hydration due to its chemo-physical properties and high pozzolanic reactivity [55,56]. Higher cement replacement with metakaolin is often hardly attractive from the economical point of view, as it is a high-price material due to its common application in other competing industries. For this reason, the suitability of other common calcined clays—not rich in kaolinite- has been investigated within the last years [57,58,59,60] and should be extended to their application in SCC as well.

Researches have investigated the behavior of binary blends of cement with RHA or calcined clays in SCC, for instance, Memon et al. [10] and Chopra et al. [31] studied the potential of RHA as VMA substitute in SCC production, and reported the possibility of a significant SCC cost reduction due to VMA elimination. Madandoust and Mousavi [34] and Ling et al. [46] explored the benefit of using metakaolin in SCC production and recommended respectively 10 and 6 wt.% cement replacement with metakaolin due to its effect on fresh properties. However, Gill and Siddique [61], and Kannan [30] have focused on ternary blends of cement with both RHA and metakaolin regarding their rheological behavior, their impact on deformation characteristics, and durability.

## 2. Research Gap

There has been a lot of research done on the influence of RHA [10,30,31] and calcined clay [32,33,34] on the fresh and mechanical characteristics of SCC. However, research on the effect of these materials on the rheology, deformation characteristics, and durability of SCC is limited especially with calcined common clays.

## 3. Properties of Rice Husk Ash (RHA) and Calcined Clays

Supplementary cementitious materials in the form of pozzolanic materials are used to replace some proportions of cement, for instance in the production of SCC. These include among others: RHA and calcined clays, both materials that obtain their pozzolanic reactivity by thermal activation (calcination). 

RHA is produced by calcination of rice husk at temperatures between 600 and 700 °C to keep the ash in the amorphous phase [31,62,63]. It contains a high amount of amorphous silica, designating it as pozzolanic material according to [64,65]. RHA consists mainly of microporous, regular, and angular particles, having large specific surface area particle micromorphology [66,67,68]. It was also characterized as having irregular, mesoporous, and rough-textured particle surfaces [31,62,69].

**Figure 1:**
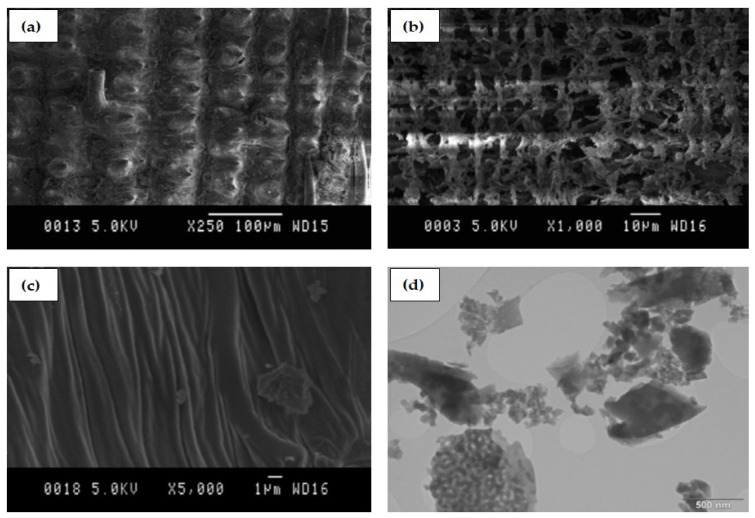
Micromorphology of rice husk ash (RHA) showing (**a**) outer surface (**b**) inter layer (**c**) Inner surface (**d**) transmission electron microscopy (TEM) image of RHA [63].

Furthermore, RHA was described as a three-layer material, containing inner and outer strata of dense structure. The interfacial strata, however, consist of a cross mesh of chips-like structure arranged in a loose honeycombed fashion containing a large number of holes (Figure 1) [63]. Transmission electron microscopy (TEM) analysis conducted on RHA calcined at 600 °C showed a very large amount of fine cooked rice-like particles as shown in Figure 1 [63]. This structure is responsible for its high specific surface area and reactivity.

Although, when obtained from an uncontrolled burning, the formation of crystalline silica is possible leading to poor pozzolanic properties and high water and superplasticizer (SP) demand [66,70]. 

Ganesanet et al. [64] observed an increase in standard consistency when RHA is added to the cement. This could be due to a higher specific surface area and or higher carbon content leading to high water demand [71]. A reduction in both the initial and final setting time of the mixture was achieved, especially at a higher replacement level of cement with RHA [64]. Le et al. [70] recorded about 79 wt.% water demand of RHA with a particle mean size of 22.6 µm which was reduced to 57 wt.% when grinding to 5.7µm mean particle size. Increasing RHA fineness has a slight effect only on its specific surface area [62].

Metakaolin (MK), as the most famous representative of calcined clays, is the metaphase of the phyllosilicate kaolinite after calcination. The calcination temperature (500–650 °C) is chosen based on the dehydroxylation temperature of kaolinite. Kaolinite is a 1:1 phyllosilicate consisting of (SiO_4_) tetrahedral and (AlO_6_) octahedral sheets that are connected via OH-groups and aluminum cations [72,73]. By dehydroxylation, the structure of metakaolin becomes highly disordered and amorphous. MK is rich in aluminum and can differ significantly in the physical properties. Besides metakaolin, the suitability of calcined common clays—with various types of 2:1 phyllosilicates and further inert components—as SCM has been investigated within the last few years [60,74,75].

The addition of up to 15 wt.% MK improved the strength of SCC [53]. At the same time Mk has a high specific surface area and high water demand. Consequently, the fluidity of self-compacting concrete decreases gradually with the increase of metakaolin content [46]. 

Table 1 shows some relevant physical and chemical properties of cement, RHA, and metakaolin reported by scholars. The cements used were ordinary Portland cement (OPC) having almost a similar range of silicon oxide of between 19.4% to 20.25%. The aluminum oxide ranges from 5.04% to 5.3%. The percentage of calcium oxide ranges between 61.2% to 63.61%. All the cements have a lost on ignition (LOI) of < 5.0. The RHA used is rich in silica ranging from 82.05% to 87.32%, with minor traces of aluminum oxide and other chemical oxides as shown in Table 1. RHA used by Zhang et al. [76] exhibits the highest LOI compared to the remaining RHAs, this could be perhaps due to the high content of unburnt carbon in the material. Metakaolin is both rich in both silica and alumina. The silicon/aluminum ratio of the metakaolin used by Kennan [77] is less than the remainder, which is an indication of purity of the metakaolin and can significantly affects its reactivity. RHA has the highest specific surface area due to its porous nature, followed by bthe metakaolin and then cement. Both RHA and metakaolin are pozzolanic in terms of chemical properties and can be used as partial substitutes to cement. 

## 4. Mix Design of Self-Compacting Concrete (SCC) with Rice Husk Ash and/or Calcined Clays

Different methods have been developed for designing SCC mixes since, unlike CVC, SCC is susceptible to nuances in material properties and other environmental factors. Initially, Okamura and Ozawa [2] used an empirical design method to achieve self-compactability, which later was adopted and modified by [80,81,82] and concrete production regulatory bodies. Both coarse and fine aggregate are kept constant using this method. The water to powder ratio and SP amount are adjusted to achieve the required degree of self-compactability. This method eliminates repeatability during SCC production. However, it is considered too complicated for practical application [5] and the water to powder ratio cannot be fixed based on strength, but rather on the self-compactability requirement.

Subsequently, methods based on rheometer tests were developed to characterize the yield stress and plastic viscosity of SCC. Sedran et al. [83] used a torsional rheometer to obtain values of yield stress and plastic viscosity to characterize SCC. RENE-LCPC^TM^ software developed based on solid suspension was used to determine the optimal packing density using less water to achieve the same or improved workability. Petersson et al. [84] developed an SCC mix design similar to the work of [83]. In their experiment, the tendency of blocking was determined using an equation to obtain the minimum paste volume while a rheometer was used to determine the suitable water to powder proportion and SP dosages. This method was adopted and modified to check the robustness of SCC produced [70,85,86,87,88]. Just like the previous method, this method does not take compressive strength as a determinant factor in designing SCC mixes and required more sophisticated tools to measure the rheology. 

Su et al. [5] used the aggregate packing method to achieve self-compactability. In this method, the least void between the loosely piled aggregate framework is determined and a liquid phase (paste) is used to fill the void and provide a lubricating layer around each particle. Although this method simplifies the SCC grades, it yields the required mix proportion for only medium to high strength concrete. This method was adopted and simplified by [70,89].

Kheder and Jadiri [90] factored in compressive strength as a determinant in designing self-compacting mixes. Their method, determines water to binder ratio based on maximum aggregate size and compressive strength requirement. Similarly, Dinakar [78,91] achieved self-compactability by considering the efficiency of pozzolanic materials added to SCC. With this method, even low-grade SCC can be achieved, although it requires adjustment to all concrete constituents in case of a minute change. Xie et al. [92] further considered even the fraction of the key oxides of a particular SCM to model both fresh and hardened properties of SCC. Their method permits achieving, both self-compactability and strength by knowing the exact characteristics of the SCC binder. 

The same mix design methods were adopted when the clinker phase is replaced with RHA and or calcined clays. Usually, a high dosage of SP is required for SCC produced with the addition of RHA and or metakaolin [10,53,55,93] due to their high surface area and water demand. The optimal replacement level for both RHA and metakaolin in SCC is usually 15 wt.% of cement [10,94,95]. Also, Dinkar and Manu [78] developed a new SCC mix design method by considering the efficiency factor of the metakaolin. Here, the replacement level is based on the efficiency factor of the metakaolin, not by simple substitution. Both RHA and MK were found to provide sufficient segregation resistance required in SCC mixes and therefore, eliminate the use of VMAs [10,34].

## 5. Fresh Properties of SCC

### 5.1. Rheological Properties of SCC

One difference between CVC and SCC is the behavior of the fresh concrete. SCC is characterized by high deformability, passing ability, and segregation resistance [2,6,96]. Various limits to these attributes are well established [97,98,99]. Commonly measured characteristic includes the flowability, viscosity, passing ability, and the segregation resistance. The flowability is divided into three classes and is determined using the slump flow diameter measured in millimeters: SF1 from 550 to 650; SF2 660 to 750; SF3 760 to 850. The viscosity is determined using V-funnel time measured in seconds and is divided into two classes: VF1, <9 and VF 2, 9 to 25. T_500_ measured in seconds can also be used to assess the viscosity of SCC and is divided into three classes: VS1, ≤2; vs. 2, 3 to 6 and VS3, >6. The passing ability is usually determined as a blocking ratio of SCC and is divided into two: PL1, ≥0.8 with two rebars; PL2, ≥0.8 with three rebars. Sieve stability is used to assess the segregation resistance of SCC. The limiting values are ≤20 for SR1 and ≤15 for SR2 [97,98,99].

Viscometers are also used to quantify SCC parameters in relation to plastic viscosity and yield values. SCC exhibits low yield stress of about 0 to 60 Pa compare to CVC and plastic viscosity of about 20 Pa∙s to almost over 100 Pa∙s. Various limits of SCC yield stress (τ) and plastic viscosity (µ) across the globe have been summarized in the form of rheographs and a workability box by [100]. The use of rheometers, as discussed under the mix design section, provide an alternative way to design and test the rheological properties of SCC in a more accurate and advanced way, and to provide the best assessment of bleeding tendency and or segregation resistance [101,102,103].

Benaicha et al. [104] proposed another simple method—V-funnel coupled to a horizontal Plexiglas channel—to assess the yield stress and plastic viscosity of SCC, especially at the construction site. A positive correlation was obtained between the theoretical yield stress and plastic viscosity obtained from the V-funnel, calculated using the equations provided by [102,105], and the actual yield stress and plastic viscosity values obtained using a R/S+ rheometer. 

### 5.2. Rheological Properties of SCC with RHA or Metakaolin (MK)

**Figure 2:**
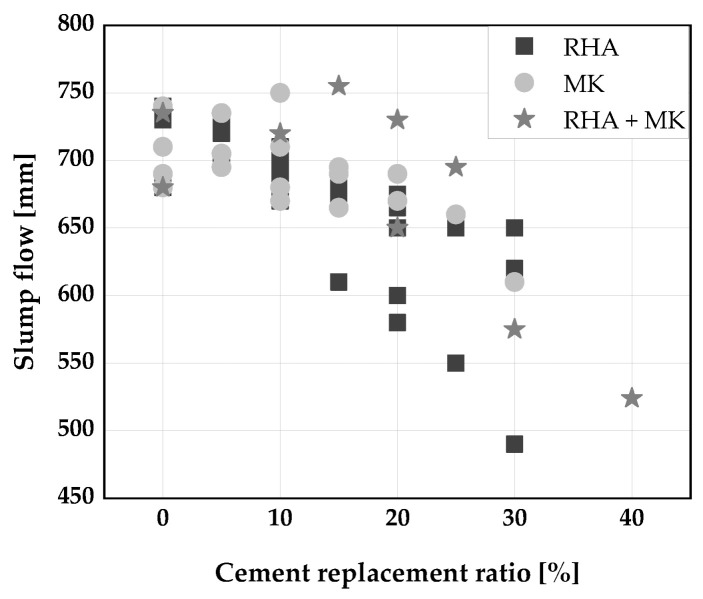
Slump flow of self-compacting concrete (SCC): Kannan [30], Chopra et al. [31], Rahman et al. [106], Madandoust [34], Gill [61].

**Figure 3:**
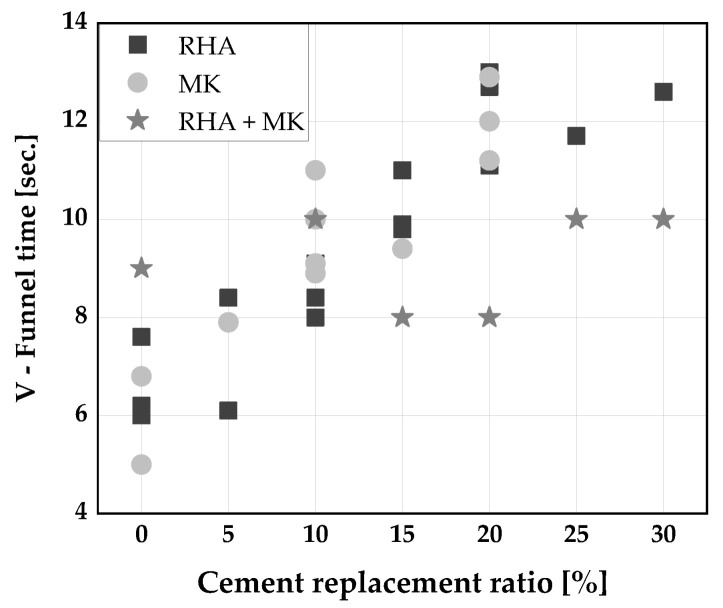
Viscosity of SCC: Chopra et al. [31], Kannan [30], Gill [61], and Madandoust [34].

**Figure 4:**
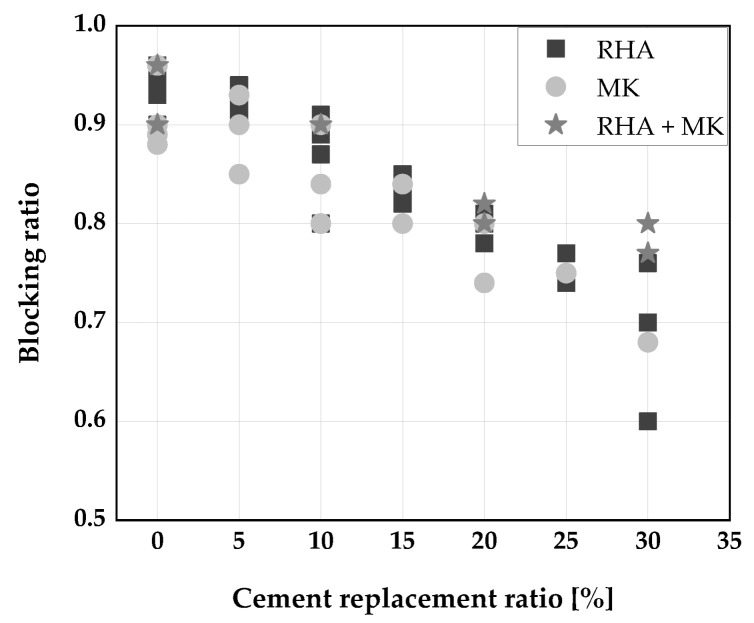
Blocking ratio of SCC: Chopra et al. [31], Kannan [30], Gill [61], Madandoust [34].

Figure 2, Figure 3 and Figure 4 display the influence of binary and ternary blends of cement, RHA, and calcined clays on fresh properties of SCC. The use of a binary blends using RHA or MK to replace cement up to 15 wt.% was found to improve the slump flow and viscosity of SCC [33,34,61,106]. The improvement in slump flow could perhaps be due to the high dosage of superplasticizer used to achieve the required properties of the fresh SCC. Consequently, a decrease in slump flow of SCC was reported [30,31] when the blends of RHA or MK were used to replaced cement. This is because both RHA and MK have high water demand compared to cement [53], leading to a decrease in the flow of SCC produced with them. At replacement levels above 25 wt.% in the case of RHA and 40 wt.% for blends of RHA and MK, the slump flow is below the limits for SCC (Figure 2). The viscosity of the SCC increased with increasing cement replacement level as shown in Figure 3. This could be because the high cohesive forces between the MK and/or RHA particles increased the friction between the mortar constituents, slowing the rate of deformation. A replacement level of 15 wt.% can be regarded more or less as threshold between VF1 and VF2. The passing ability of SCC keeps decreasing with an increase in cement replacement by RHA and MK. Any cement replacement beyond 15 wt.% with RHA and MK resulted in a poor passing ability of the SCC produced [30,34,61] (Figure 4). On the other hand, a good passing ability was obtained, at 30 wt.% cement replacement with a ternary blend of RHA and MK [30,61]. 

Rheological measurements on self-compacting mortar produced with RHA as clinker replacement revealed increased yield stress and viscosity due to an increase in water demand [62]. Ling et al. [46] reported similar findings related to the use of metakaolin as SCM.

Although both RHA and MK have good potential to be used as SCM in concrete production due to their superior pozzolanic reactivity, their use as clinker substitute in SCC production results in a significant decrease in slump flow and an increase in viscosity. Consequently, when both materials are to be added to SCC higher dosage of SP is required.

## 6. Hardened Properties of SCC

### 6.1. Compressive Strength

**Figure 5:**
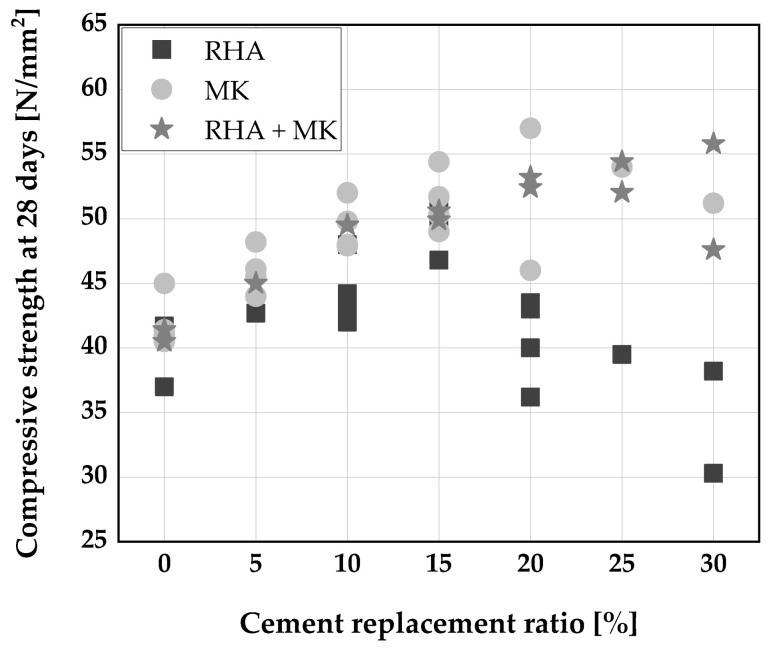
Compressive strength of SCC: Chopra et al. [31], Kannan [30], Kavitha [53], Madandoust [34], Vivek [107], and Gill [61].

RHA addition up to 10 wt.% of binder was found to increase the compressive strength of SCC [10]. The authors used RHA as a substitute to VMA and not as SCM. The increase in strength is due to a reduction in water to binder ratio, because of RHA addition resulting in denser particle packing, pore, and grain size refinement [10]. When used as a SCM, RHA improves the microstructure of the paste matrix and transition zone, due to its high reactivity leading to the formation of more C–S–H, thereby improving the strength development of SCC [30,31,106]. Similarly, a compact formation of hydration product leading to a reduction in porosity of the concrete was observed [69] when RHA replaced up to 15 wt.% of cement. Figure 5 shows the compressive strength of SCC produced with RHA and MK and in combination as cement replacement.

It becomes clear from Figure 5 that cement replacement with RHA up to 15 wt.% can improve compressive strength of SCC [30,31]. Also, a decrease in compressive strength was reported by [106] for all the replacement levels with RHA, which could perhaps be due to the extent of treatment (calcination and grinding) performed on the RHA which largely determines its reactivity [76,108]. Le et al. [70] reported a decrease in compressive strength at the early age of curing for a higher percentage of cement replacement with RHA. It was followed by an increase in compressive strength at 56 days of curing and above. Furthermore, RHA is considerably more effective than fly ash in improving compressive strength, mainly due to its high content of reactive amorphous silica and higher specific surface area [70].

Metakaolin can lead to an acceleration of cement hydration and strength development when properly treated and added to cement [109]. An increase in compressive strength was reported by [34,107] when up to 15 wt.% cement is replaced with MK as depicted in Figure 5. A higher replacement level just maintains compressive strength but yield no further gain.

Ternary blends of RHA and MK were found to improve both early and later compressive strength development in SCC as reported by [61,110]. A higher percentage replacement level of 20 wt.% of cement and above was possible for optimum performance when the two pozzolanic materials were blended.

### 6.2. Ultrasonic Pulse Velocity

Ultrasonic pulse velocity (UPV) is a proper parameter for testing the homogeneity and integrity of concrete non-destructively. Increased UPV values correspond to the densification in the internal structure of the SCC [111]. Higher UPV values were achieved at all ages of SCC when replacing 20 wt.% of cement with RHA [111].

**Figure 6:**
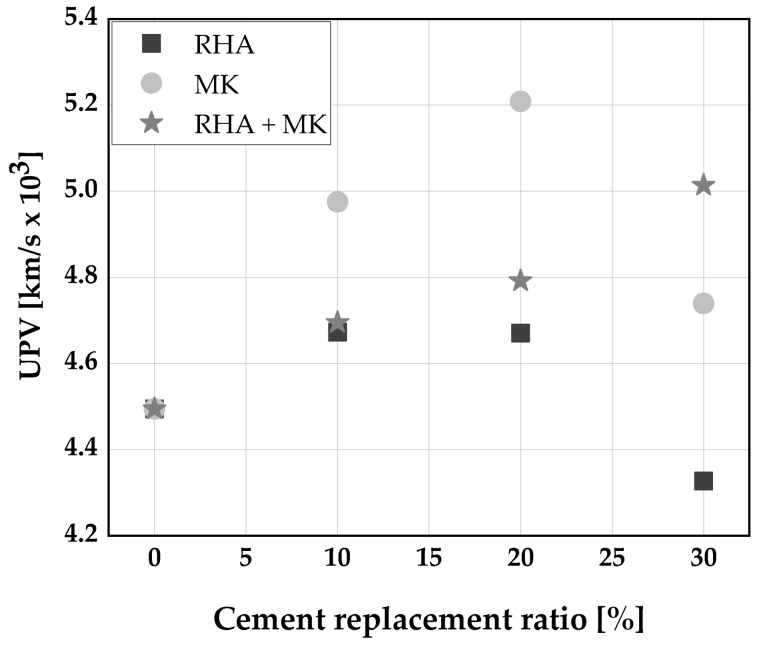
Ultrasonic pulse velocity of SCC [30].

Özcan and Kaymak [109], observed a strong positive relationship between compressive strength and UPV values of SCC produced with a binary blend of cement and metakaolin. This is an indication that whenever metakaolin is replacing cement up to an optimal level, the homogeneity, and integrity of the SCC increases. Similarly, Kannan [30] reported a “good” concrete quality for SCC produced without the addition of pozzolanic materials, while an “excellent” concrete quality was reported for SCC with cement replaced with up to 25 wt.% RHA; “excellent” concrete quality was achieved for all levels of cement replacement with MK and a ternary blend of RHA and MK as shown in Figure 6. The concrete integrity classification was based on IAEA, 2002 [112] classification as “excellent”, above 4.5 km/s; “good”, 3.5 to 4.5 km/s; “doubtful”, 3.0 to 3.5 km/s; “poor”, 2.0 to 3.0 km/s and “very poor”, below 2.0 km/s.

### 6.3. Modulus of Elasticity

**Figure 7:**
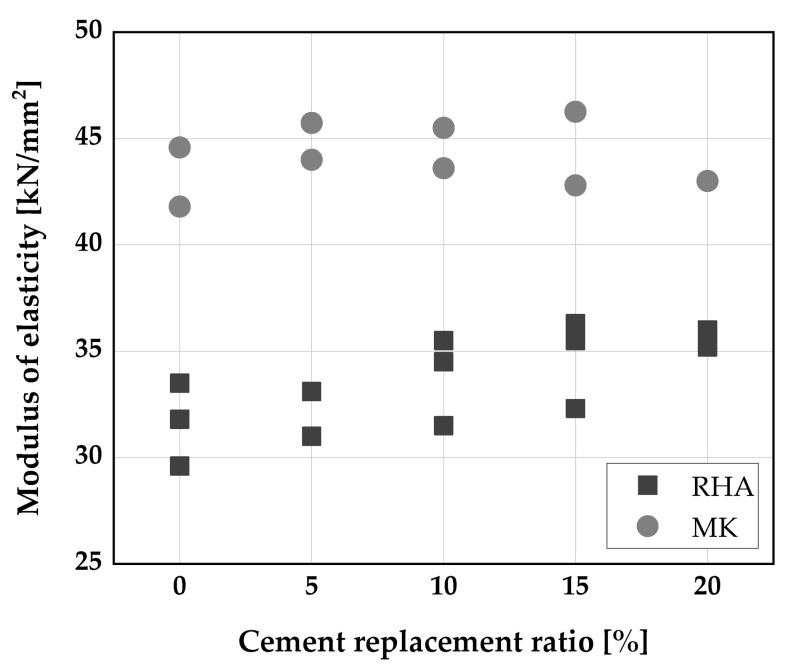
Modulus of elasticity: Molaeiraisi [116], He [117], Ramezanianpour et al. [118], Barkat et al. [22], Johari [119].

The mechanism of achieving SCC entails a reduction in size and quantity of coarser aggregate content of the mixture, this raised concern that SCC may have lower values of modulus of elasticity, which in turn will affect the deformation characteristics of the concrete [25]. Several publications investigated the influence of RHA and metakaolin addition on the modulus of elasticity properties of SCC [30,46,113,114]. Metakaolin had no significant impact SCC [22,56,93,115] as displayed in Figure 7. A slight increase in modulus of elasticity was observed when RHA was used as SCM [116,117,118]. A further increase in the replacement level with RHA beyond 10 wt.% of cement caused rather a slight reduction in the modulus of elasticity values of SCC [116] (Figure 7).

### 6.4. Shrinkage and Creep

Shrinkage and creep behavior are important attributes to be studied when introducing any pozzolanic material as SCM. This is related to high water evaporation rates, especially in dry conditions, due to slow hydration kinetics resulting in high free plastic shrinkage compared to SCC without SCMs [120]. So far, there has been no literature on shrinkage and creep behavior of SCC with RHA and metakaolin in ternary blends.

**Figure 8:**
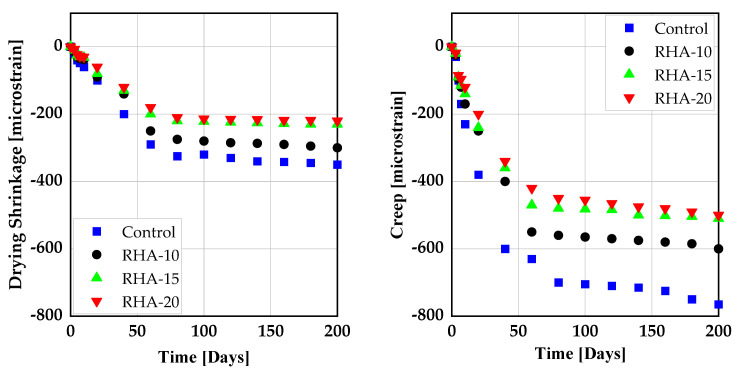
Drying shrinkage and creep of concrete produced with RHA: He [117].

Few papers deal with the impact of RHA on shrinkage behavior of SCC [121,122]. Both reported no significant effect due to the addition of RHA on the shrinkage behavior of SCC. However, when finely ground, RHA increased the drying shrinkage of concrete due to its microfine particles [123]. However, when used as a substitute to the clinker phase in CVC, RHA was found to reduce the drying shrinkage of concrete [66,124]. This might be due to the ability of RHA to behave like an internal curing agent, whereby continuously releasing the absorbed free water in its mesoporous cellular structure for hydration, and thus controlling concrete internal relative humidity, delaying self-desiccation and preventing autogenous shrinkage of concrete [68,118,125,126]. Similarly, creep and drying shrinkage strains were found to exhibit a similar tendency. Concrete specimens with a high RHA to binder ratio yield a smaller strain and creep coefficient compared to the control specimens as shown in Figure 8 [117].

**Figure 9:**
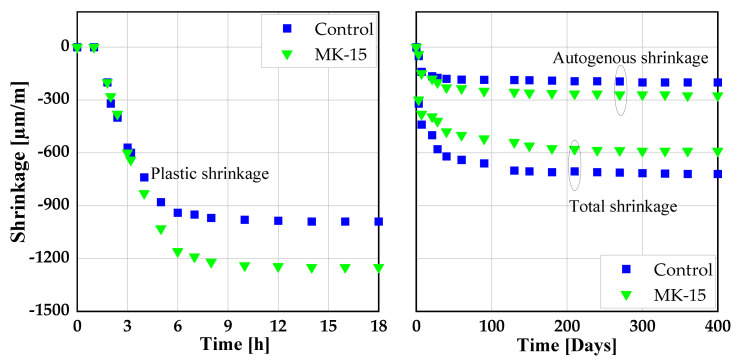
Plastic, autogenous and total shrinkage of concrete produced with metakaolin (MK) [120].

With the addition of MK at 15 wt.%, the drying plastic shrinkage value of SCC increased by about 20 % since MK contributes to the high water evaporation rate of the system and consequently the tendency of higher sensitivity to drying [120,127]. SCC specimens with 15 wt.% MK as cement substitute experienced high values of autogenous shrinkage up to 10 days of curing, beyond which the shrinkage values decline and become less than the control specimens as depicted in Figure 9 [120]. This effect of MK is due the formation of finer pores leading to more self-desiccation and consequently high autogenous shrinkage [120,128,129]. On the other hand, SCC produced with MK as SCM exhibited lower drying and autogenous shrinkage values than the control specimens [120,130]. This was attributed to low evaporation of the free water and the pozzolanic reactivity of MK leading to the formation of denser microstructure and hence higher resistance to deformation [33,131]. The autogenous and drying shrinkage remains less than the SCC specimens without MK [120].

**Figure 10:**
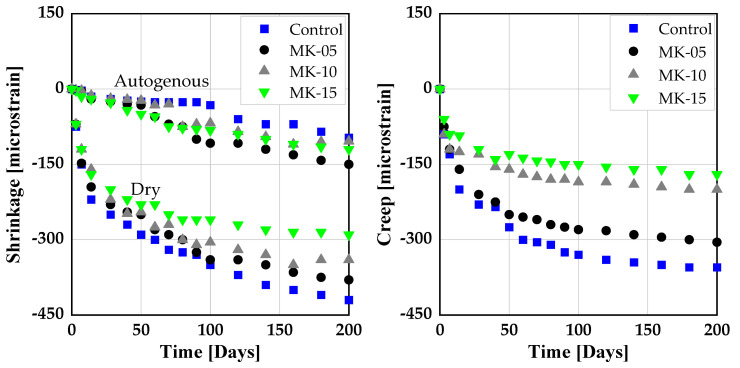
Shrinkage and creep of conventional vibrated concrete (CVC) produced with MK [120].

Nesvetaev et al. [132], studied the effect of metakaolin on the creep properties of SCC. On control specimens, they observed an increased final creep coefficient (Δ 1.3 to 1.8) of SCC compared to NVC. However, when MK and silica fume were both added to the mixture of SCC, the creep coefficient dropped by 0.5 to 0.6 compared to CVC. From this point of view, it is important to study the effect of RHA with and without MK on shrinkage and creep behavior of SCC. Figure 10 shows the shrinkage and creep of CVC produced with MK as cement substitute. Autogenous as well as drying shrinkage of CVC is significantly smaller than observed for SCC.

## 7. Durability Properties

### 7.1. Water Absorption and Sorptivity

**Figure 11:**
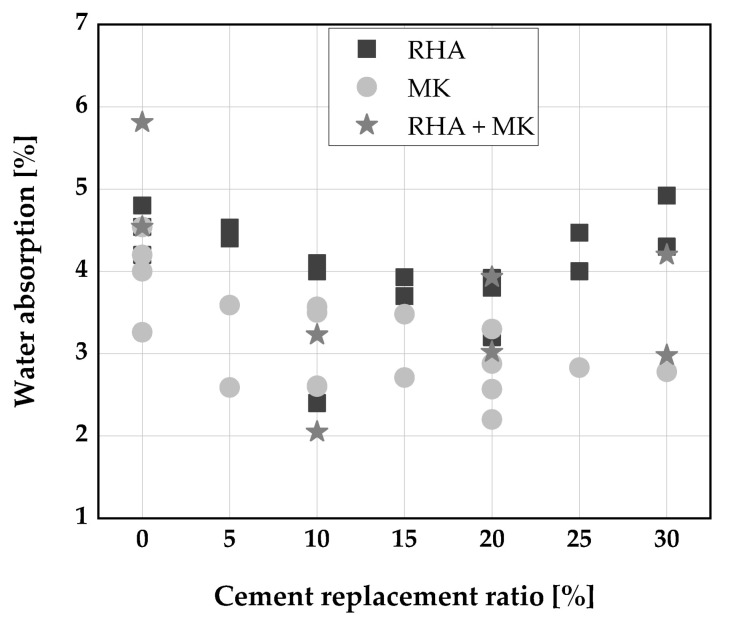
Water absorption of SCC produced with RHA and MK: Kannan [110], Rahman [106], Madandoust [34], Gill [135].

Figure 11 shows the water absorption of SCC produced with a binary blend of RHA, MK, and a ternary blend of RHA and MK. A good concrete quality [133] having low water absorption capacity was reported when RHA, MK, and blends of RHA and MK were added to SCC. A decrease in water absorption for RHA replacement up to 20 wt.% was reported by [110,134]. This decrease is due to additional C-S-H formation caused by the pozzolanic reaction of RHA and a micro filler effect leading to refinement of the grading of the concrete [110]. However, Rahman et al. [106] reported an increase in water absorption of SCC at all replacement levels when uncontrolled burnt RHA was used to replace cement.

Similarly, a reduction in water absorption of SCC was noticed for all replacement levels (5, 10, 15, and 20 wt.%) of MK as shown in Figure 11. This is due to a reduced porosity and finer pore size distribution [32,34,110,134]. Gill/Siddique [135] reported a sharp decrease in water absorption when a ternary blend of RHA and MK was used to replace cement at 15 wt.%, while a further increase in replacement level leads to an increase in water absorption. However, Kannan/Ganesan [110] and Gill/Siddique [135] reported a continuous decrease in water absorption up to 30 wt.% cement replacement level with a ternary blend incorporating RHA and MK. Generally, water absorption values of SCC made with these ternary blends are significantly reduced compared to the binary systems with RHA only.

Sorptivity of SCC produced without the addition of SCMs was found to be very high compared to CVC and was attributed to the higher binder content of SCC [107]. However, the addition of MK up to 30 wt.% reduced sorptivity and lower capillary suction [107]. Kannan and Ganesan [110], reported a reduction by 7% when 15 wt.% of RHA was used as cement substitute, while a further increase in the replacement level resulted in a rise of the sorptivity values. Similar to the water absorption, a higher reduction in sorptivity (26%) was observed when the ternary blend (15 wt.% RHA and 15 wt.% MK) was used to replace cement compared to the binary blends only [110].

### 7.2. Porosity

**Figure 12:**
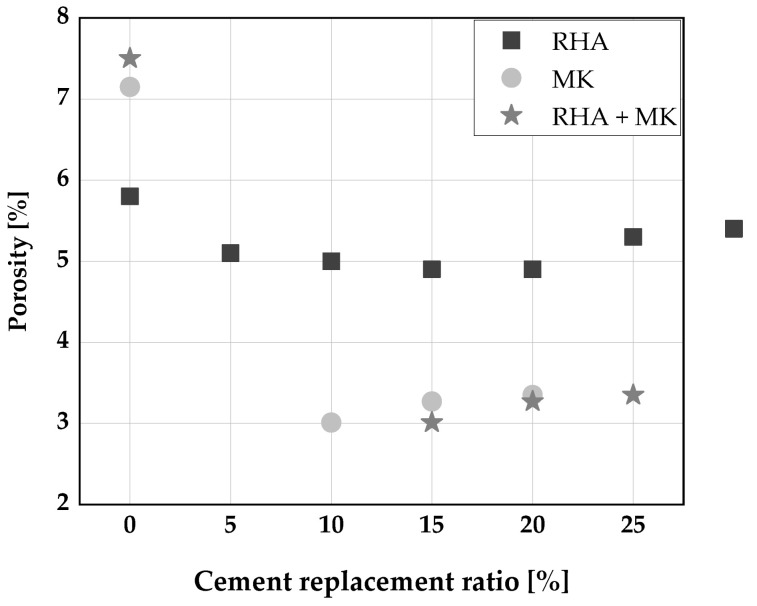
Porosity of SCC produced with RHA and MK: Chopra et al. [31], Barkat et al. [22], Gill [135].

The use of RHA (up to a replacement level of 25 wt.% of cement) was found to decrease the porosity of SCC and, thereby, to lower its permeability [31,136]. This decrease becomes more pronounced with an increase in curing age as shown in Figure 12. Similarly, metakaolin, up to 25 wt.% cement replacement slightly decreased the porosity of SCC [22,137]. However, these findings hold only up to 28 days age of concrete. A minimum porosity was achieved for RHA and MK at a replacement level of. At a later age, Gill [135] reported an increase in porosity especially with higher percentages of cement replacement exceeding 15 wt.% with RHA and MK, and attributed this to the higher surface area of RHA, and its subsequent water demand.

### 7.3. Chloride Penetration Resistance

**Figure 13:**
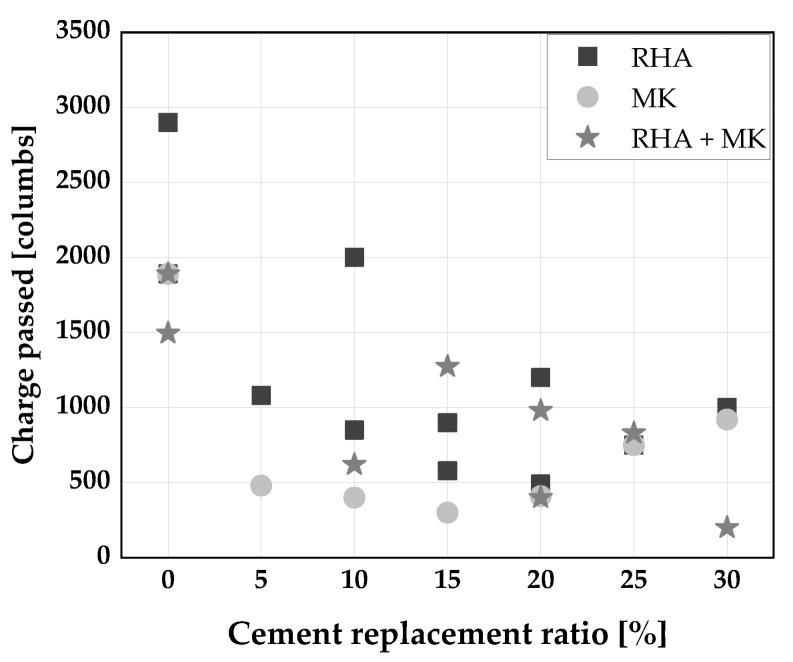
Rapid chloride penetration of SCC produced with RHA and/or MK [110].

The rapid chloride permeability test (RCP), by passing an electric charge through concrete specimens, is used to assess the chloride penetration resistance of concrete according to (ASTM C1202 [138], DIN EN 12390-11 [139], etc.). Figure 13 depicts the results of RCP of SCC with RHA, MK, and a blend of RHA and MK as SCM. A reduction in total electrical charge passed through SCC was observed by [31,110,140] when RHA was used as a cement substitute. This reduction is due to the densification of the SCC microstructure, because of the pozzolanic reaction of RHA leading to the formation of more C-S-H in the system and consequently a reduction of micropores and cracking tendency [31].

Similarly, for all cement replacement with MK up to 15 wt.%, a reduction in chloride permeability was reported by [53]. Badogiannis [141] expressed chloride permeability in terms of chloride migration coefficient and observed about a 70% decrease in permeability when MK was used to replace cement. This is perhaps due to the densification of the pore structure, from the pozzolanic reactivity of MK, leading to the reduction in the width of the interfacial transition zone by creating more C–A–H, thereby decreasing the diffusion rate of SCC [53]. Also, the least total charge passed by RCP was recorded by [110] and [135] with a ternary blend of RHA and MK at up to 40 wt.% cement replacement. This indicates that a ternary blend of RHA and MK provides a better chloride penetration resistance than the individual binary blend with RHA and MK, due to further densification of the pore structure and the formation of more C-S-H gel [135,142].

### 7.4. Resistance to Magnesium Sulfate

Sulfate resistance of SCC with RHA and MK is also an aspect of durability that only a few researchers [53,135,142] have paid attention to. Kavitha and Shanthi [53] studied the weight loss, compressive strength, and micro-structure of SCC with MK, up to 12 weeks of curing in 5 wt.% MgSO_4_ solution and reported a greater resistance at all replacement levels with MK compared to control SCC. This was attributed to the pozzolanic reaction between CH and MK, leading to pore size refinement, and increased resistance to diffusion of harmful ions [53]. SCC with MK replacing cement up to 20 wt.% lost less weight in a MgSO_4_ solution than the remaining specimens [53].

**Figure 14:**
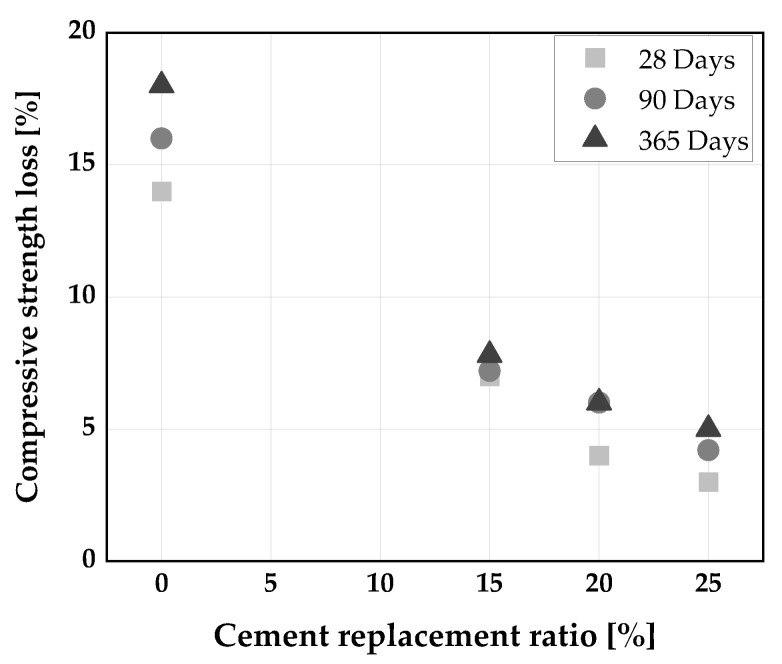
Compressive strength loss of SCC [135].

In addition, Gill and Siddique [135] have reported a reduction in compressive strength of SCC produced with a ternary blend of RHA and MK even at 28 days of curing in the sulfate environment. SCC produced with the addition of ternary blend (10 wt.% MK + 10 wt.% RHA) showed better resistance to the magnesium sulfate attack compared to the remaining specimens as shown in Figure 14. The mechanism behind the resistance to MgSO_4_ is perhaps because of the ternary blend of RHA and MK leading to more C-S-H in the mixture, thereby leading to pore size refinement, and increase resistance to diffusion of harmful ions [135].

### 7.5. Carbonation

Carbonation occurs due to the reaction between carbon dioxide and the alkaline components of cement hydration, mainly CH. This causes a reduction in the pH-value of the paste pore solution [143]. The addition of RHA as a substitute to the clinker phase in concrete decreased the amount of CH on the one hand but provided better resistance to carbonation at the same time [144,145,146,147]. Lower carbonation coefficients were reported when RHA was used to replace cement at 20 wt.% and, a further decrease was obtained when 1 wt.% K_2_SO_4_ was added to the mixture as a chemical activator [144]. Consequently, the use of RHA increases the depth of carbonation in concrete [148,149]. This was attributed to lower cement content in the system and higher porosity [118] allowing more CO_2_ to penetrate into the concrete. This could perhaps be due to the treatment given to the RHA or because the pore solution under the carbonation process is yet to be consolidated, as the result of the accelerated method adopted [146]. The authors were not aware of literature on the effect of RHA on the carbonation resistance of SCC.

Metakaolin as partial replacement of cement was found to be more effective in reducing the carbonation resistance of SCC, than observed for CVC [150]. In both cases, the use of MK led to a reduced carbonation depth and improved the permeability resistance. This was due to the consumption of CH and pore size refinement from the pozzolanic reactivity of MK. Similar results were reported by [151,152]. On the other hand, a slight decrease of pH values compared to the control specimens was observed when MK was used to substitute cement at 10 wt.% and subjected to 14 years of natural carbonation [146].

### 7.6. Freeze-Thaw

**Figure 15:**
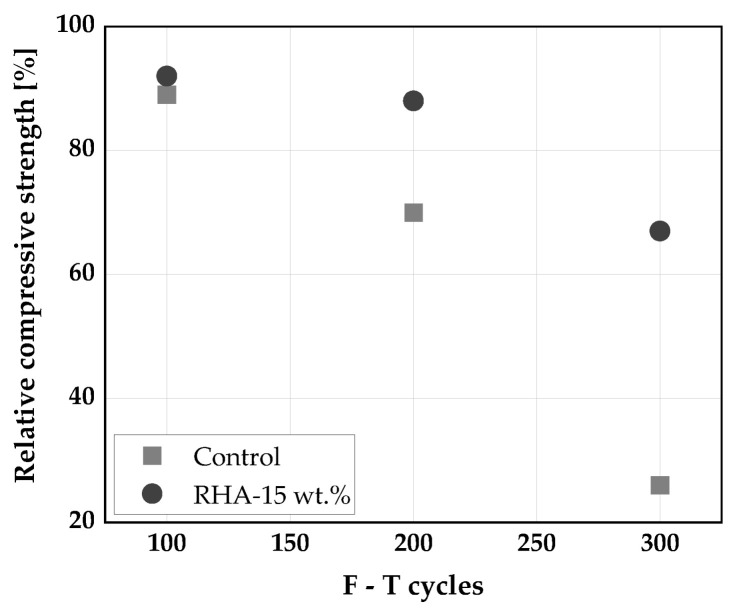
Relative compressive strength of SCC subjected to F-T [153].

The use of RHA to replace cement decreases the internal damage caused by freeze-thaw (F-T) and as well, limits its impact on the dynamic modulus of elasticity of SCC subjected to F-T cycles. The durability factor, determined based on ASTM C 666-15 method of SCC without RHA subjected to up to 300 F-T (+4 to −18 °C and subsequently −18 to +4 °C for 5 h) cycles was found to be 56%. When RHA was used as cement replacement at 15 wt.%, the durability factor increased to 80% [153]. SCC with cement replacement suffered less weight and compressive strength losses, its electrical resistivity increased, and exhibited higher values of dynamic modulus of elasticity when subjected to F-T cycles compared to their companion control specimens [153]. This was explained by the consumption of CH by the reactive silica in RHA and producing more C-S-H in the cement matrix, leading to the formation of dense microstructure and thereby decreases porosity and permeability of the SCC [147]. Similar observations hold for CVC [154,155]. Figure 15 shows the relative compressive strength of SCC subjected to 100, 200, and 300 F-T cycles and at +4 to −18 °C and, subsequently, −18 to +4 °C for 5 h.

Duan et al. [156] observed a reduction of the interconnected pores in the concrete structure when MK was used as cement replacement. This prevented osmotic pressure resulting from the migration of supercooled water and thereby improved the F-T resistance of the concrete. The reduction of the interconnected pores is attributed to better particle packing and pore size refinement in the course of the pozzolanic reaction of MK [156,157]. An improvement in the residual UPV values, compressive strength, and weight loss was observed when MK was used as cement replacement in concrete subjected to F-T cycles by [157,158,159]. The authors found no data on the combined effect of RHA and MK on the impact of F-T.

## 8. Discussion

Both RHA and metakaolin are pozzolanic and are used as a SCM for SCC production [10,30,31,32,33,34]. RHA derives its pozzolanicity from optimization of proper calcination and grinding [31,62,63]. The hydration mechanism of RHA in concrete is related to the consumption of portlandite and enhancement of precipitation of C-S-H, which reduces the specific pore size of the binder matrix and densified its microstructure [70,71,76]. These attributes make it possible to partially substitute cement with RHA up to 20 wt.% without an adverse effect on its strength and or durability [30,31,106]. Another possible reason for this could be caused by the ability of RHA to behave like an internal curing agent, whereby it is continuously releasing the absorbed free water from its mesoporous cellular structure for hydration, which facilitates the precipitation of more C-S-H in the system [68,118,126]. Metakaolin, on the other hand, behaves similarly to RHA in binder matrix densification but the mechanism of its pozzolanic reactivity is more complex. Metakaolin reacts faster than RHA and promotes in addition to the silicate the aluminate reaction especially in the presence of CaCO_3_, thereby promoting the precipitation of hemi/monocarboaluminate AFm phases of binder hydrates [22,30,115]. Cement can be partially replaced with up to 45 wt.% of a blend of metakaolin and limestone (LC^3^, cement) without an adverse effect on its strength and durability. The use of both RHA and metakaolin as a partial replacement for cement increases the water demand of the blended system [21,46,123] and, therefore, care has to be taken in selecting the degree of viscosity of the self-compactability required especially at higher levels of cement replacement. Pure metakaolin has other industrial attraction and is more costly compared to cement. Although the suitability of other common clays not rich in kaolinite has been established as discussed in [60,74,75], research on their use as binary blend with cement, or as multi-blend in addition to RHA, is deficient especially in the aspects of rheological measurements, shrinkage, creep and the durability of SCC. Therefore, the need to expand the frontier of research on studying the rheology, mechanical and durability of SCC produced with calcined common clays and its multi-blend with RHA is paramount.

## 9. Conclusions

The following conclusions were drawn from the findings of the study:

- Metakaolin was found to be the most commonly used calcined clay for the production of SCC thus far. The suitability of other clayey materials such as calcined clay waste, calcined red mud, and calcined common clays with low kaolinite content for the production of SCC was not yet explored.
- Rice husk ash and metakaolin are pozzolanic materials and have the potentials to be used as supplementary cementitious materials in self-compacting concrete production. The potentials of rice husk ash as a viscosity-modifying agent in concrete production is also established.
- Both rice husk ash and metakaolin have high water demand compared to cement. Therefore, their use as a supplementary cementitious material in SCC entails the application of a high dosage of superplasticizers.
- Rice husk ash was found to be very effective in increasing self-compacting concrete strength due to its high content of reactive amorphous silica and higher specific surface area. Metakaolin, on the other hand, was also found to improve early strength development due to its fast pozzolanic reaction.
- The optimal replacement level for both the binary blend with RHA and MK is usually between 15 and 20 wt.%. However, with a ternary blend with RHA and MK, larger cement replacements of between 20 to 40 wt.% are possible.
- Studies on the effect of ternary blends of rice husk ash and metakaolin on shrinkage and creep behavior of SCC were not found by the authors.
- Although researchers have presented results of the durability of SCC produced with a blend of rice husk ash and metakaolin, there is a need to further study the effect of these materials on porosity, carbonation and freeze-thaw resistance of SCC.
- The review disclosed a lack of knowledge when it comes to the use of low-kaolinitic calcined clays as sole SCM or together with rice husk ash which could be a very promising combination for e.g., several countries in Africa.
- Greenhouse gas emissions and environmental problems could be reduced by partially replacing cement with RHA and metakaolin.

## 10. Proposal for Further Investigation

The effect of RHA on the rheological properties of SCC in terms of yield stress, plastic viscosity, shear-thickening behavior, and workability retention was reported in the literature. However, such detailed studies were not found on a binary blend with calcined common clays nor a ternary blend of RHA and calcined common clay. The fracture mechanism and durability (in terms of freeze-thaw, carbonation, chemical resistance etc.) of SCC produced with RHA and calcined common clays as a partial replacement for cement needs to be further investigated as well.

## Figures and Tables

**Table 1:** Properties of cement, RHA, and metakaolin.

Materials	Reference	SiO_2_ (%)	Al_2_O_3_ (%)	Fe_2_O_3_ (%)	CaO (%)	MgO (%)	Na_2_O (%)	K_2_O (%)	LOI (%)	Specific Surface Area (m^2^/g)	Mean Particles Size (µm)
Cement	[77]	20.21	5.06	3.18	63.30	4.20	0.09	0.52	3.07	316 ^Bl^	23.40
	[64]	20.25	5.04	3.16	63.61	4.56	0.08	0.51	3.12	326 ^Bl^	22.50
	[62]	19.4	5.3	2.5	61.2	1.2	0.07	0.61	4.9	2.07 ^B^	7.07
RHA	[77]	82.05	0.45	2.21	0.62	0.62	0.95	4.43	4.97	916 ^Bl^	6.57
	[64]	87.32	0.22	0.28	0.48	0.28	1.02	3.14	2.10	36.47 ^B^	3.80
	[77]	87.20	0.15	0.16	0.55	0.35	1.12	3.68	8.55	38.90 ^B^	7.00
Metakaolin	[77]	49.50	44.23	0.92	0.17	0.08	0.10	0.02	0.32	2342 ^Bl^	3.71
	[78]	54.3	38.3	4.28	0.39	0.08	0.12	0.50	0.68	15.00 ^B^	NR
	[79]	56.20	37.20	1.40	1.20	0.20	NR	1.20	2.10	18.70 ^B^	11.50

NR = Not Reported; Bl = Blain’s (m^2^/kg); B = BET (m^2^/kg).

## Data Availability

Data Sharing is not applicable.
